# An Association Between Job Stress and Poor Social Support Among Healthcare Workers in Northeastern Malaysia

**DOI:** 10.7759/cureus.38937

**Published:** 2023-05-12

**Authors:** Wira Alfatah Ab Aziz, Kamarul Imran Musa, Mohd Ismail Ibrahim, Yelmizaitun Osman, Mohd Nazri Shafei

**Affiliations:** 1 Private Medical Practice Control, Kelantan State Health Department, Kota Bharu, MYS; 2 Department of Community Medicine, Universiti Sains Malaysia School of Medical Sciences, Kota Bharu, MYS; 3 Environmental and Occupational Health Unit, Kelantan State Health Department, Kota Bharu, MYS

**Keywords:** malaysia, social support, healthcare workers, job stress, proportion

## Abstract

Introduction: Job stress is an important occupational health problem globally. Hence, identification of workers at risk of developing job stress is paramount to the decision-makers. This study aims to estimate the proportion of job stress and its relationship with different categories of healthcare workers (HCWs) in the primary care and public health settings in northeastern Malaysia.

Methodology: A cross-sectional study involving 520 HCWs across all categories was conducted in Kelantan State, Malaysia. A proforma and validated Malay version of the Job Content Questionnaires were administered to obtain the data. The participants were then classified into four categories of workers according to Karasek’s job demands-control model classification which were active, passive, high strain, and low strain.

Results: We found that a total of 145 (28.5%) HCWs in the study have job stress (high-strain job type). HCWs with a degree or higher qualification had the highest proportion of job stress (41.2%), while the diploma group has the lowest proportion of job stress among the four academic qualification groups (22.9%). Pearson chi-square shows a significant association between Karasek’s job types and the level of social support from their supervisors (p < 0.05) but no association between job strain and the level of supervisor's social support (p > 0.05).

Conclusion: Job stress among HCWs is prevalent, and the professional group had the highest percentage of risk job stress as compared to other groups. There is a significant association between the supervisor’s social support and Karasek's job strain categories.

## Introduction

Healthcare services in Malaysia are delivered via a dual system, namely, the public and private sectors. Under the Ministry of Health, the public sector offers tax-funded healthcare, while out-of-pocket payments mostly finance the private sector. In terms of primary healthcare services, private clinics outnumber public health clinics by a 6:1 ratio. However, public health clinics manage a far more significant number of patients daily with a median attendance rate of 111.5 presentations per day as compared to 33.0 per day in private clinics [1].

Job stress is defined as the harmful physical and emotional responses that occur when the requirements of the job do not match the capabilities, resources, or needs of the worker [2]. It results from the interaction of the worker and the conditions of work. Views differ, however, on the importance of worker characteristics versus working conditions as the primary cause of job stress, and it was to be explained by various theoretical models [3]. These differing viewpoints are critical because they suggest different ways to prevent stress at work [4].

One of the prominent theories of job stress was proposed by Robert Karasek’s job demands-control model. The popularity of the job demands-control model is most likely due to its simplicity, the ease with which it can be tested empirically, and the practical implications that can be divined from this model. The basic premise of the job demands-control model is that the most stressful or ‘high strain’ jobs are those in which employees are subjected to high levels of demands, yet at the same time have very little control over their work [5]. The main idea behind the job demands-control model is that job control buffers the impact of job demands on the strain. It can help enhance employees’ job satisfaction with the opportunity to engage in challenging tasks and learn new skills [6].

Multiple surveys conducted by different centers worldwide on the prevalence of job stress found that it ranges from 26% to 40% according to the CDC [2]. A local study among assistant medical officers found that more than 13% of them had experienced stress [7]. On top of that, a considerable proportion of pharmacists have a moderate level of job satisfaction to the extent that it has a significant relationship with work-life balance, which leads to poor performance [8,9].

Job stress has been shown to affect the employee’s performance by reducing productivity, increasing job insecurity, and leading to wider health issues [10,11]. In the healthcare setting, the negative effects of job stress are diverse and affect both individuals and organizations [11]. There has been a lack of studies published related to job stress among healthcare workers (HCWs) in Malaysia, even though understanding and knowing the magnitude of job stress is important for this country that is becoming a high-income-status country. Findings from such studies will provide the latest snapshot of the level of job stress among HCWs in Malaysia and the management in the Malaysian healthcare system to develop future policies in the healthcare setting that would be able to reduce job stress. This is especially true when the Malaysian government is promoting KOSPEN Plus since 2016. KOSPEN is an abbreviation for "Komuniti Sihat Pembina Negara" Program in Malay which can be translated as Nation Builder Healthy Community Program. KOSPEN Plus is the extension of the program to cater for intervention of non-communicable disease prevention at workplaces. The objective of the KOSPEN Plus programme is to nurture healthy and productive employees in a safe working environment [12].

In this study, we aim to understand job stress issues in greater detail among HCWs working in public health settings in Kelantan, Malaysia. Specifically, we seek to estimate the overall prevalence and the prevalence of job stress based on different categories of HCWs. We would also examine the association between job stress and the perceived level of support from both supervisors and co-workers from HCWs’ point of view.

## Materials and methods

A cross-sectional study was conducted among HCWs working in the primary care and district health office in Kelantan, Malaysia, from January to June 2019. Despite the multi-ethnicities found among the residents, the Malay population appears to be predominant, which accounted for more than 95% of the total population [13]. A total of 536 HCWs aged 40 to 60 years were required for the study. We decided to select that age group (40-60 years) of workers as our government strongly recommended them to undergo annual health screening. To ensure an equal proportion of samples across all categories, we applied a systematic proportionate random sampling to select each worker based on the registries from each selected health clinic and district health office. The list of workers from the selected clinic was obtained from the human resource. Then, the proportion of workers in each clinic was calculated with the total number of workers in all the selected clinics as the denominator. Based on the proportion, the required sample size for each clinic was determined and a systematic random sampling was then applied to select the participant for the study. Participants with a previous history of mental illness (based on personal declaration) and who had worked less than six months at the current posting were excluded from the study.

A study proforma and the Job Content Questionnaire (JCQ), which is a self-administered instrument designed to measure the social and psychological characteristics of jobs, were applied in the study. The JCQ has been validated and translated into more than 29 languages [14]. It was developed based on Karasek’s job demands-control model and was widely used as a reliable tool to measure job stress among workers around the globe.

The selected HCWs were required to fill in the validated Malay version Job Content Questionnaire (M-JCQ) (Copyright R. Karasek JCQ Center Global ApS) as the primary tool for this study [15]. The M-JCQ consists of four main domains, with a total of 41 items. The domains measured in this tool are decision latitude, psychological job demands, physical job demands and social support. Each of the items has four choices on a Likert scale: strongly disagree, not agree, agree, or strongly agree. The score for the M-JCQ was calculated according to formulas for job content instrument construction based on the guidelines from the Job Content Questionnaire and User’s Guide (Revision 1.8 10/15 revised policy) [14]. The overall distribution of HCWs was classified based on Karasek's job types which were active, passive, low strain, and high strain. Those who belonged to only the high-strain group were defined as having job stress, which meant that they had low job decision latitude in combination with high job demand. The M-JCQ licence for the study was approved by the JCQ Centre Global (License Number: 52828081111).

Statistical analyses were performed by using Jamovi version 1.2.0 and IBM SPSS Statistics for Windows, Version 26 (Released 2019; IBM Corp., Armonk, New York, United States). We began with the descriptive analysis of the sociodemographic characteristics of the participants followed by analyzing the scoring distribution of the JCQ scales, which consists of four quadrants (job types), as follows:

1. High-strain jobs (high psychological demands and low levels of control)

2. Low-strain jobs (low psychological demands and high levels of control)

3. Active jobs (high psychological demands and high/sufficient control over job activities)

4. Passive jobs (low psychological demands and low levels of control)

The scores were generated based on the formula provided in the JCQ and User’s Guide (Revision 1.8 10/15 revised policy). The median of the total score was calculated and marked as the cut-off point to distinguish between the low and high categories [14].

To highlight the impact of the job category on the scores, we described further the results in different categories based on the highest education attained. We also examined the proportion of Karasek’s job types based on the four quadrants. Finally, we analyzed the relationship between Karasek’s job types and the level of social support from their supervisors and co-workers.

## Results

A total of 520 participants were involved in this study with a response rate of 97%. Table 1 shows that the mean (SD) age of male and female HCWs was 47.8 (5.07) and 44.9 (4.22) years, respectively. A total of 333 (64%) of them were female. It was also found that the HCWs from the professional group accounted for only 3.2% of the total participants. Most of the workers were non-smokers (91.9%). A total of 440 (85.4%) of them were overweight, and 92 (18.2%) had hypertension. 

**Table 1:** Characteristics of the participants SBP: Systolic blood pressure DBP: Diastolic blood pressure

Variables		n (%)
Sex		
	Male	187 (36.0)
	Female	333 (64.0)
Academic qualification		
	Degree or higher	17 (3.2)
	Diploma	209 (40.4)
	Senior high school	186 (35.9)
	Junior high school	106 (20.5)
Smoking status		
	Non-smoker	477 (91.9)
	Smoker	42 (8.1)
Body mass index		
	Normal (<22.9)	76 (14.6)
	Pre-obese (23.0–274)	211 (40.9)
	Obese I (27.5–34.9)	194 (37.7)
	Obese II and above (>35)	35 (6.8)
Blood pressure status		
	Normal (SBP <130 and/or DBP <85)	310 (61.2)
	At risk (SBP 130-139 and/or DBP 85-89)	104 (20.6)
	Hypertension Stage 1 (SBP 140–159 and/or DBP 90-99)	74 (14.6)
	Hypertension Stage 2 and above (SBP >160 and DBP >100)	18 (3.6)
Known diabetes mellitus		
	Yes	47 (9.0)
	No	473 (91.0)

Table 2 shows the distribution of the scores according to the JCQ domains. The decision latitude score was calculated from a total of nine questions that gave us a wide range of scores from 42 to 88 with the mean (SD) score of 64.5 (6.33) apart from the formula for job content instrument construction. The domain for psychological job demand has a score ranging from 8 to 31, with a mean (SD) of 18.1 (4.11). By contrast, physical job demand has a minimum score of 5 and a maximum score of 20 with a mean (SD) score of 12.4 (2.16). The social support domain has a range score of 4 to 16 for supervisor support and 8 to 16 for co-worker support with a mean (SD) of 11.7 (1.70) and 12.2 (1.30), respectively, for each domain.

**Table 2:** Distribution of the scores according to the job content questionnaires domain among participants

Domain	Minimum score	Maximum score	Mean (SD) score
Decision latitude	42	88	64.5 (6.33)
Skill discretion	22	48	34.0 (3.37)
Decision authority	12	44	30.5 (4.86)
Psychological job demands	8	31	18.1 (4.11)
Physical job demands	5	20	12.4 (2.16)
Physical exertion	3	12	7.9 (1.56)
Physical isometric loads	2	8	4.6 (1.17)
Supervisor social support	4	16	11.7 (1.70)
Co-worker social support	8	16	12.2 (1.30)

Table 3 shows the distribution of the scale categories among the HCWs stratified by their highest educational attainment. For the scale of decision latitude, which is the essential measurement for the authority to make a decision, it was found that the HCWs with a degree or higher qualification had a slightly lower mean score than those with diploma qualification, but it was higher than those with senior and junior high school qualification. The psychological job demand score, which is another essential scale to determine job stress, shows that HCWs with a degree or higher qualification had the lowest mean score. The diploma and senior high school categories shared a similar average score of 18.2. There was a similar average score for physical job demands across all categories ranging from 12.3 to 12.6. The mean score for the social support of the supervisor showed a decreasing score across education categories (highest among degree or higher qualification and lowest among junior high school) but had almost a similar score for social support among co-workers.

**Table 3:** Distribution of job content questionnaire scales by the highest educational level attained

	Highest educational level attained											
Scale	Degree or higher (n = 17)			Diploma (n = 208)			Senior high school (n = 186)			Junior high school (n = 106)		
	Min score	Max score	Mean score (SD)	Min score	Max score	Mean score (SD)	Min score	Max score	Mean score (SD)	Min score	Max score	Mean score (SD)
Decision latitude	52	76	64.5 (5.32)	52	82	65.1 (6.03)	42	88	64.2 (6.71)	50	78	64 (6.42)
Skill discretion	28	40	33.6 (3.1)	26	42	34.2 (3.21)	22	44	34 (3.39)	22	48	33.8 (3.69)
Decision authority	24	36	30.8 (3.68)	20	44	30.8 (4.59)	12	44	30.1 (5.16)	16	40	30.2 (4.99)
Psychological job demands	12	22	17.1 (2.86)	9	29	18.2 (4.1)	8	31	18.2 (4.03)	9	31	18 (4.46)
Physical job demands	10	16	12.5 (1.62)	5	20	12.6 (2.28)	7	20	12.3 (2.12)	6	18	12.4 (2.05)
Physical exertion	6	10	8.12 (1.45)	3	12	7.92 (1.6)	3	12	7.85 (1.61)	4	11	7.8 (1.44)
Physical isometric loads	4	6	4.35 (0.79)	2	8	4.64 (1.19)	2	8	4.45 (1.11)	2	8	4.59 (1.29)
Supervisor social support	11	14	12.3 (0.77)	4	16	11.8 (1.76)	4	16	11.6 (1.73)	6	16	11.5 (1.62)
Co-worker social support	11	15	12.2 (0.81)	9	16	12.3 (1.78)	9	16	12.3 (1.29)	8	16	12.1 (1.43)

The HCWs with high strain made up the highest proportion, which accounted for 28.5% of them, followed by the active job type (27.9%). Meanwhile, the proportion of those with low strain and passive job types was 21.8%. Table 4 shows the distribution of the Karasek job types based on their highest educational level attained. It was found that the professional group (degree holder or higher) had the highest percentage of high strain as compared to other groups. Other Karasek categories showed quite a fair distribution across all educational levels except for the passive group where there was only one (5.9%) HCW from the professional group.

**Table 4:** Karasek’s job type and highest educational level attained

Karasek’s job type	Degree or higher n (%)	Diploma n (%)	Senior high school n (%)	Junior high school n (%)
Active	5 (29.4)	67 (32.7)	42 (23.2)	26 (25.0)
Low strain	4 (23.5)	45 (22.0)	41 (22.7)	21 (20.2)
Passive	1 (5.9)	46 (22.4)	41 (22.7)	23 (22.1)
High strain	7 (41.2)	47 (22.9)	57 (31.4)	34 (32.7)

Table 5 shows the association between Karasek’s job type and social support. The social support was divided into two elements, which were from their supervisor and from their co-workers. There was a statistically significant association between Karasek’s job type and the level of the supervisor’s social support. However, there was no significant association between Karasek’s job type and the level of co-workers’ social support.

**Table 5:** Association between job stress and social support using Chi-squared test * Pearson chi-square statistics

Karasek’s job type	Supervisor social support		X^2^ stat (df)	p-value*	Co-workers social Support		X^2^ stat (df)	p-value*
	High	Low			High	Low		
Active	111(81.6)	25(18.4)	10.26	0.016	29(20.7)	111(79.3)	6.398	0.094
Low strain	75(69.4)	33(30.6)			33(29.7)	78(70.3)		
Passive	71(64.5)	39(35.5)			20(18.7)	87(81.3)		
High strain	107(75.4)	35(24.6)			25(17.5)	118(82.5)		

Finally, we proceed with the determination of the relationship between job stress (high strain) and the highest educational level attained using Pearson chi-square analysis. We found that there was no statistical significance difference between job stress and the highest educational level attained (Table 6).

**Table 6:** Association between job stress and highest educational level attained

	Highest educational level attained				X^2^ stat (df)	p-value
	Degree or higher n (%)	Diploma n (%)	Senior high school n (%)	Junior high school n (%)		
Job Stress						
Yes	7 (4.8)	47 (32.4)	57 (39.3)	34 (23.4)	6.27 (3)	0.100
No	10 (2.8)	158 (43.8)	124 (34.3)	69 (19.1)		

## Discussion

This study looked at the proportion of job stress and the relationship with the highest educational level and social support among HCWs in primary care and public health settings in Kelantan, Malaysia. The study found that there was no association between job stress and the highest education attained. The present study found that job stress is associated with supervisor social support but not with co-worker social support.

The HCWs had alarmingly many risk factors for potentially suffering from non-communicable diseases in the future. The prevalence of obesity among HCWs is significantly higher than that among the Kelantan general population at 60%, based on data from the National Health and Morbidity Survey 2019, and the Malaysian general population, which was at 50.1% [16]. Another study conducted in Perak among HCWs found that 49.9% of HCWs were overweight or obese, similar to the national prevalence [17]. According to Avila et al. (2015), obesity not only is linked to physical health outcomes but also has been extensively associated with mental illness [18].

The present study found that the authority of decision-making (shown by the decision latitude score) among HCWs with at least a degree qualification was no better than that among HCWs who held diplomas. This may happen as explained by the model of the psychosocial class structure proposed by Karasek (1989) because both nurses and doctors in Malaysian healthcare settings will join the top management positions with significant decision-making commitment [6].

It was found that both the active and high-strain job types had a higher proportion than other categories. The proportion of job stress (high-strain job type) among primary HCWs was higher than that of job stress among university staff in Malaysia [19]. The reason could be due to the nature of their job whereby HCWs often deal with life-and-death situations on a regular basis. The high stakes and the responsibility associated with saving lives can create significant emotional and psychological pressure. They are also responsible for the well-being and care of patients, which can involve complex decision-making, multitasking, and managing multiple cases simultaneously. In addition, the nature of their work requires them to display empathy while maintaining a level of professional detachment, which can be emotionally challenging. While university staff members may also face stressors such as heavy workloads, research pressures, and administrative responsibilities, the nature and intensity of these stressors can differ from those encountered by HCWs. It is important to recognize that stress levels can vary among individuals within each profession, and factors such as personal resilience, workplace culture, and support systems also play significant roles in determining overall stress levels. Meanwhile, a study in Taiwan among healthcare professionals found a lower proportion of job stress, which was 16.5 % [20]. Difference in the job description and scope in other countries may explain the difference in the proportion of job stress between countries.

Despite the fact that the social support scale was not being used directly to identify job stress, it was included in the M-JCQ for several reasons. The addition of social support as the third dimension in job stress was meant to expand the existing theory of Karasek’s job demands-control model [14]. According to Chen et al. (2020), social support may have significant mediating effects on the link between job stress, depression and anxiety [21]. Our findings signified the strong association between all Karasek’s categories and the level of supervisor social support.

Finally, educational levels did not have any significant association with job stress. In other words, job stress could happen to any of the job categories. However, the model of a psychosocial class structure suggested that the more prestigious occupations, such as professions and nursing, would typically lie in the active job quadrant [14]. This finding highlighted the importance of regular screening for mental health among primary HCWs in Malaysia. Since the inception of health promotion in the workplace by KOSPEN Plus, it has been made compulsory for all the HCWs to undergo the annual medical examination for those aged 40 and above including mental health screening.

While the JCQ has its strengths, it also has certain limitations that should be taken into consideration. Among the limitations of the Karasek's JCQ is that it measures job stress using a limited number of items and scales, focusing primarily on job demands, control, and social support. It may not capture the full complexity of work-related stressors and their impact on individuals. The JCQ also relies on self-reporting, meaning that respondents provide their own perceptions and interpretations of their job characteristics and stress levels. This can introduce bias and potential inaccuracies, as individuals may have different perceptions of their work environment and stress levels. Lastly, the JCQ primarily focuses on psychosocial factors related to work stress, such as job demands and control. It does not comprehensively cover other important aspects of work stress, such as organizational factors, work-life balance, interpersonal conflicts, and career development issues.

For a future study, we recommend using a more objective assessment for job stress. This can involve observing work conditions, interactions, and behaviors. Interviews can also be conducted to gather more in-depth information about individuals' experiences of stress at work. Physiological measures such as heart rate variability, cortisol levels, and blood pressure can provide objective indicators of stress. However, these measures often require specialized equipment and expertise. On top of that, conducting a job analysis which involves examining the specific tasks, responsibilities, and demands of a job may help to identify potential stressors such as excessive workload, time pressures, physical demands, or exposure to hazardous conditions.

## Conclusions

There is a relatively high proportion of HCWs in Kelantan suffering job stress. The supervisor’s social support does have an association with Karasek’s categories but not co-workers’ support. The findings from this study highlighted the importance of implementing regular medical surveillance among HCWs, particularly in mental health, which is often overlooked. The current policy only required those aged 40 and above to undergo the health screening, which started with the implementation of KOSPEN Plus in 2016.
